# Association between *ATM* gene polymorphisms, lung cancer susceptibility and radiation-induced pneumonitis: a meta-analysis

**DOI:** 10.1186/s12890-017-0555-7

**Published:** 2017-12-15

**Authors:** Zhipeng Yan, Xiang Tong, Yao Ma, Sitong Liu, Lingjing Yang, Xin Yang, Xue Yang, Min Bai, Hong Fan

**Affiliations:** 1 Department of Respiratory Medicine and Critical Care Medicine, West China Hospital/West China School of Medicine, Sichuan University, Guoxuexiang 37, Chengdu, 610041 China; 2 Department of Internal Medicine, No.4 West China Teaching Hospital, Sichuan University, Renming South Road 3rd Section 18, Chengdu, 610041 China; 3 Department of Respiration, East Branch, Sichuan Provincial People’s Hospital, Sichuan Academy of Medical Science, No. 585 Honghe North Road, Chengdu, 610110 China

**Keywords:** Ataxia telangiectasia mutated, Polymorphism, Lung cancer, Radiation-induced pneumonitis, Meta-analysis

## Abstract

**Background:**

Previous studies have suggested that DNA double-strand break (DSB) repair is an important protective pathway after damage. The ataxia telangiectasia mutated (ATM) gene plays an important role in the DNA DSB repair pathway. DNA damage is a major cytotoxic effect that can be caused by radiation, and the ability to repair DNA after damage varies among different tissues. Impaired DNA repair pathways are associated with high sensitivity to radiation exposure. Hence, *ATM* gene polymorphisms are thought to influence the risk of cancer and radiation-induced pneumonitis (RP) risk in cancer patients treated with radiotherapy. However, the results of previous studies are inconsistent. We therefore conducted this comprehensive meta-analysis.

**Methods:**

A systematic literature search was performed in the PubMed, Embase, China National Knowledge Internet (CNKI) and Wanfang databases to identify studies that investigated the association between the *ATM* gene polymorphisms and both lung cancer and RP radiotherapy-treated lung cancer (the last search was conducted on Dec.10, 2015). The odds ratio (OR) and 95% confidence interval (CI) were used to investigate the strength of these relationships. Funnel plots and Begg’s and Egger’s tests were conducted to assess the publication bias. All analyses were performed in STATA 13.0 software.

**Results:**

Ten eligible case-control studies (4731 cases and 5142 controls) on lung cancer susceptibility and four (192 cases and 772 controls) on RP risk were included. The results of the overall and subgroup analyses indicated that in the *ATM* gene, the rs189037 (−111G > A, −4519G > A), rs664677 (44831C > T, 49238C > T) and rs664143 (131,717 T > G) polymorphisms were significantly associated with lung cancer susceptibility (OR = 1.21, 95% CI = 1.04–1.39, *P* = 0.01; OR = 1.26, 95% CI = 1.06–1.49, *P* = 0.01; OR = 1.43, 95% CI = 1.15–1.78, *P* < 0.01). Additionally, the rs189037 variant was significantly associated with RP risk (OR = 1.74, 95% CI = 1.02–2.97, *P* = 0.04). No publication bias was found in the funnel plots, Begg’s tests or Egger’s tests.

**Conclusions:**

The results indicate that the *ATM* rs189037, rs664677 and rs664143 gene polymorphisms are risk factors for lung cancer, while the *ATM* rs189037 variant was significantly associated with RP risk.

## Background

Lung cancer is the most common cancer and the most common cause of death from cancer worldwide [1]. Although smoking is a major risk factor for lung cancer, only 15% of smokers develop lung cancer [2, 3], suggesting that different populations are more or less susceptible to carcinogens and that genetic factors probably play an important role in cancer aetiologies [4].

Radiotherapy is an important treatment for cancers. However, 10% to 20% of cancer patients who undergo radiotherapy develop severe radiation-induced pneumonitis (RP), which influences their quality of life. Approximately half of patients with RP die [5–7]. RP is a common dose-limiting toxicity of radiotherapy [8]. Its risk factors include patient-related factors, such as gender, smoking and pulmonary function, and treatment-related factors, such as the radiation dose and irradiated lung volume, and whether surgery or chemotherapy was performed [9–11]. However, these factors do not sufficiently explain the wide variations observed in susceptibility among patients. Recent studies have shown that variation in individual susceptibility to cancers and RP is affected by gene polymorphisms, especially those affect DNA repair [12].

The ataxia-telangiectasia mutated (ATM) gene is one such DNA repair gene. *ATM* plays an important role in the repair of DNA damage, especially DNA double-strand breaks (DSBs) [13, 14]. DSBs can lead to genetic information loss, harmful gene variations and chromosomal rearrangements, which can result in the development of cancer [15]. The ATM protein is a phosphoinositide 3-kinase (PI-3 kinases) [13]. Once an exogenous injury, such as ionizing radiation, induces DSBs, the ATM protein is activated via autophosphorylation. It can then phosphorylate dozens of downstream substrates, many of which are key factors in DNA repair, apoptosis, cell cycle arrest and gene regulation [16, 17]. The affected genes include the checkpoint protein RAD50, cycle checkpoint kinase (CHK2), tumor suppressor P53, breast cancer protein 1 (BRCA1), the repair protein Nijmegen breakage syndrome 1 (NBS1), and the oncogenic protein murine double minute 2 (MDM2) [18, 19]. Polymorphisms in *ATM* may influence the structure and function of the protein, leading to defects in the activation of cell cycle checkpoints, DNA DSB repair, and cell apoptosis. Additionally, *ATM* gene mutations may alter the radiosensitivity of cells [8, 20, 21]. The development of cancers and radiation- induced side effects, including pneumonitis, is often linked to these abnormal cells [20, 22].

Previous studies have shown that several *ATM* gene polymorphisms (e.g., rs664143, rs664677, rs189037, and rs609429) may be associated with susceptibility to lung cancer [23–25], and that others (e.g., rs189037) may be associated with RP risk [26, 27]. However, the results of those studies have been inconsistent, and previous meta-analyses were not comprehensive. Therefore, we conducted this comprehensive meta-analysis to evaluate the association between *ATM* gene polymorphisms and both susceptibility to lung cancer and the risk of RP in lung cancer patients treated with radiotherapy. To the best of our knowledge, the current study is the most comprehensive analysis of the relationship between susceptibility to lung cancer and *ATM* gene polymorphisms and the first meta-analysis to evaluate the association between RP risk and *ATM* gene mutations.

## Methods

### Eligible studies

We searched the PubMed, Embase, China National Knowledge Internet (CNKI) and Wanfang databases to identify studies that investigated the association between *ATM* gene polymorphisms and lung cancer as or RP in lung cancer patients treated with radiotherapy (the last search was performed on Dec.10, 2015) using the following search terms: “ATM” and “cancer” or “lung cancer” or “Lung Neoplasms” and “polymorphism” or “variant”; “ATM” and “radiation pneumonia” or “radiation pneumonitis” and “polymorphism” or “variant”. The references listed in the resulting articles were also searched to identify additional relevant articles.

The following were the inclusion criteria for studies in our meta-analysis: (1) case-control studies focused on *ATM* polymorphisms and lung cancer susceptibility or RP risk, (2) data on genotype frequencies were available for both the cases and controls, (3) published in English or Chinese, and (4) the genotype distribution of the control group was in accordance with Hardy-Weinberg equilibrium. The following exclusion criteria were applied: (1) no control group, (2) duplication of a previous study, and (3) no usable data on genotype frequency.

### Data extraction

Two authors independently extracted the data from all eligible publications. The following information was extracted from each relevant study: the first author’s name, country of origin, publication year, ethnicity of the study individuals, cancer type, genotyping methods, sample size and genotype frequencies.

### Statistical analysis

All statistical analyses were performed by Stata 13.0. The strength of the association between an *ATM* polymorphism and lung cancer or RP was measured by odds ratios (ORs) with 95% confidence intervals (CIs). Heterogeneity was evaluated by the chi-squared (x^2^) and I-squared (I^2^) test. If *P* > 0.10 and I^2^ < 50%, no heterogeneity was detected among the studies, and the OR was calculated by the fixed-effects model. Otherwise, the random-effects model was used. To evaluate ethnicity-specific effects, a subgroup analysis was performed by ethnicity group. The potential publication bias was assessed by funnel plots, Begg’s test and Egger’s test.

## Results

### Characteristics of the studies

**Fig. 1:**
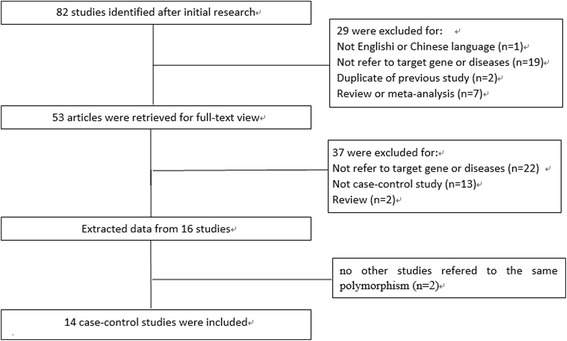
Flow diagram of the selection process

A total of 82 studies were initially identified from different databases. After reading their titles and abstracts, we excluded 29 reviews or meta-analyses that were not relevant to our study or not published in English or Chinese. After the full-text versions were read, we further excluded 37 studies that did not offer usable data (such as genotype or allele frequency) or other essential information. We extracted information from 16 studies. Two of these studies were excluded because no other studies referred to the same polymorphism (for example, there was only one study on the association between the *ATM* gene rs373759 and susceptibility to lung cancer), and they were therefore not suitable for inclusion in this meta-analysis. Finally, 14 studies were included in our study (Fig. 1). Among these, 10 studies [21, 24, 25, 28–34] included 4731 cases and 5142 controls, evaluated four single nucleotide polymorphisms (SNPs), and were focused on the association between *ATM* gene polymorphisms and susceptibility to lung cancer. Four studies [22, 26, 27, 35] included 192 cases and 772 controls, evaluated one SNP, and focused on the association between the risk of RP and *ATM* gene polymorphisms. Additionally, two studies [33, 35] provided only the total number of common genotypes (e.g., AA and AG or AG and GG). Hence, we calculated results for only one model (AA vs. AG + GG). The characteristics of the included studies are summarized in Table 1 and Table 2.

**Table 1:** Characteristics of studies included in our meta-analysis

First Author	Year	Country	Ethnicity	Case/control	Method
On lung cancer susceptibility					
Xia W [28]	2010	China	Asians	264/264	TaqMan
Bi N [29]	2014	China	Asians	254/260	PCR-RFLP^a^
Lo YL [25]	2010	China	Asians	730/730	MassARRAY
Shen L [21]	2014	China	Asians	487/516	TaqMan
Hsia TC [30]	2013	China	Asians	358/716	PCR-RFLP
Deng Q [31]	2010	China	Asians	315/315	PCR^b^
Kim JH [24]	2006	Korea	Asians	616/616	PCR
Liu J [32]	2014	China	Asians	852/852	TaqMan
Yang H [33]	2007	USA	Caucasians	556/556	TaqMan
Landi S [34]	2007	Germany	Caucasians	299/317	PCR
On RP risk					
Zhang L [22]	2010	China	Asians	44/209	PCR
Xiong H [26]	2012	China	Asians	56/305	PCR-RFLP
Xiao Y [27]	2015	China	Asians	48/49	TaqMan
Yang M [35]	2011	China	Asians	44/209	PCR

^a^PCR-RFLP, polymerase chain reaction-restriction fragment length polymorphism

^b^PCR, polymerase chain reaction

**Table 2:** Distributions of *ATM* gene polymorphisms allele and genotypes in different groups

rs189037	AA(case/control)	AG(case/control)	GG(case/control)	*P* value
Lo YL [25]	145/124	345/354	238/239	0.72
Shen L [21]	99/92	240/272	148/152	0.12
Hsia TC [30]	64/122	176/339	118/255	0.61
Kim JH [24]	105/113	316/306	190/195	0.71
Liu J [32]	200/154	435/434	217/264	0.29
rs664677	TT(case/control)	TC(case/control)	CC(case/control)	*P* value
Bi N [29]	40/42	120/128	94/88	0.69
Lo YL [25]	126/104	346/356	257/268	0.41
Kim JH [24]	82/71	294/303	233/230	0.06
Landi S [34]	108/85	134/170	47/54	0.05
Yang H [33]	445^a^/441^a^		102/99	
rs664143	AA(case/control)	AG(case/control)	GG(case/control)	*P* value
Xia W [28]	86/68	139/139	39/55	0.30
Bi N [29]	71/73	137/128	46/53	0.82
Deng Q [31]	102/86	164/158	49/69	0.82
Kim JH [24]	180/185	356/312	87/119	0.54
Yang H [33]	104/95	433^b^/441^b^		
rs609429	CC(case/control)	CG(case/control)	GG(case/control)	*P* value
Lo YL [25]	142/125	349/369	232/234	0.32
Landi S [34]	35/52	101/113	92/70	0.62
Yang H [33]	103/100	436^c^/438^c^		
rs189037(RP)	AA(case/control)	AG(case/control)	GG(case/control)	P value
Zhang L [22]	8/38	26/89	10/82	0.12
Xiong H [26]	24/74	22/149	10/82	0.70
Xiao Y [27]	11/13	22/22	15/14	0.48
Yang M [35]	34^d^/127^d^		10/82	

^a^this figure represents the sum of TT and TC

^b^this figure represents the sum of AG and GG

^c^this figure represents the sum of CG and GG

^d^this figure represents the sum of AA and AG

*P* values represent the results of Chi-square tests of Hardy - Weinberg equilibrium

### Meta-analysis results

**Fig. 2:**
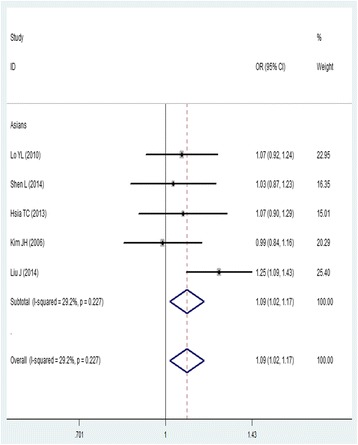
Forest plot of the association between rs189037 and lung cancer risk (A vs. G). OR: odds ratio, CI: confidence interval

For the *ATM* rs189037polymorphism, 3043 cases and 3430 controls from five case- control studies on lung cancer susceptibility were included in the present meta-analysis. The experimental populations in all five studies were Asian. We found that *ATM* rs189037 polymorphism A allele was associated with an increased risk of lung cancer (AA versus AG/GG, OR = 1.16, 95% CI = 1.03–1.32; AA versus GG, OR = 1.21, 95% CI = 1.04–1.39; A versus G, OR = 1.09, 95% CI = 1.02–1.17) (Fig. 2; Table 3). No publication bias was detected in a funnel plot or Egger’s test (*P* = 0.323).

**Table 3:** Total results from different comparative genetic models

Gene polymorphisms	OR	95% CI	*P*	I^2^(%)	Models
On lung cancer risk					
rs189037					
AA + AG vs. GG	1.10	0.99–1.22	0.08	8.7	Fixed
AA vs. AG + GG	1.16	1.03–1.32	0.02	19.9	Fixed
AA vs. GG	1.21	1.04–1.39	0.01	32.7	Fixed
AG vs. GG	1.06	0.95–1.19	0.30	0	Fixed
A vs. G	1.09	1.02–1.17	0.01	29.2	Fixed
rs664677					
TT + TC vs. CC	1.01	0.89–1.14	0.88	0	Fixed
TT vs. TC + CC	1.26	1.06–1.49	0.01	1.1	Fixed
TT vs. CC	1.20	0.98–1.46	0.08	0.0	Fixed
TC vs. CC	0.96	0.83–1.11	0.60	0.0	Fixed
T vs. C	1.08	0.98–1.18	0.12	9.7	Fixed
rs664143					
AA + AG vs. GG	1.43	1.18–1.73	0	0	Fixed
AA vs. AG + GG	1.10	0.95–1.27	0.20	0	Fixed
AA vs. GG	1.43	1.15–1.78	<0.01	0	Fixed
AG vs. GG	1.43	1.17–1.75	<0.01	0	Fixed
A vs. G	1.15	1.04–1.28	<0.01	0	Fixed
rs609429					
AA vs. AG + GG	0.97	0.72–1.32	0.85	59.3	Random
On RP risk					
rs189037					
AA + AG vs. GG	1.72	1.18–2.52	<0.01	2.6	Fixed
AA vs. AG + GG	1.34	0.67–2.66	0.57	57.4	Random
AA vs. GG	1.74	1.02–2.97	0.04	35.8	Fixed
AG vs. GG	1.48	0.92–2.37	0.11	23.2	Fixed
A vs. G	0.57	0.18–1.77	0.33	97.5	Random

**Fig. 3:**
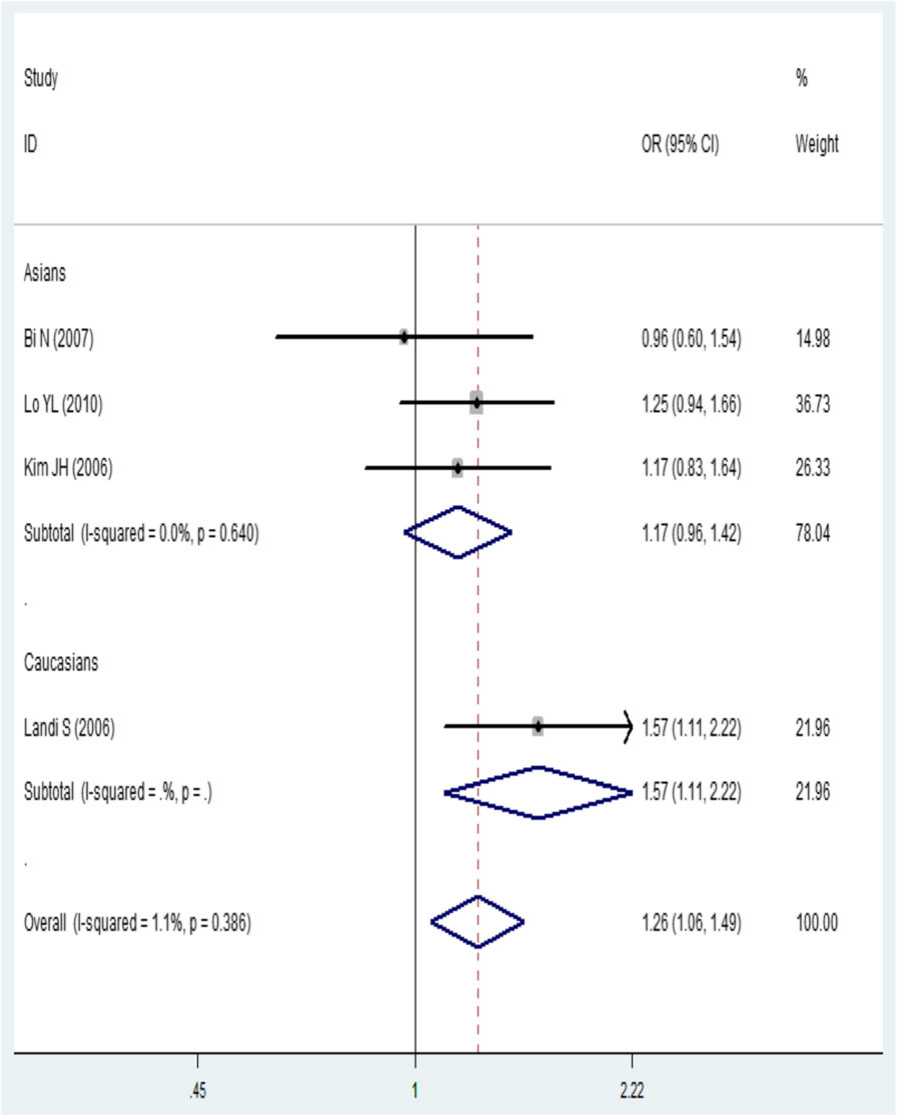
Forest plot of the association between rs664677 and lung cancer risk (TT vs.TC + CC). OR: odds ratio, CI: confidence interval

For *ATM* rs664677, five studies containing 2428 patients and 2439 controls were included. We found that the TT genotype was associated with a significantly higher risk of lung cancer (TT versus TC/CC, OR = 1.26, 95% CI = 1.06–1.49), and no significant association was found for this genotype in other genetic models (Fig. 3; Table 3). There was no significant association in the subgroup analysis of Asian populations. No publication bias was detected in a funnel plot or Egger’s test (*P* = 0.565).

For *ATM* rs664143, 1983 cases and 1981 controls from five studies were included. We found that the A allele was associated with an increased risk of lung cancer (AA/AG versus GG, OR = 1.43, 95% CI = 1.18–1.73; AA versus GG, OR = 1.43, 95% CI = 1.15–1.78; AG versus GG, OR = 1.43, 95% CI = 1.17–1.75; A versus G, OR = 1.15, 95% CI = 1.04–1.28). No publication bias was detected in a funnel plot or Egger’s test (*P =* 0.303).

**Fig. 4:**
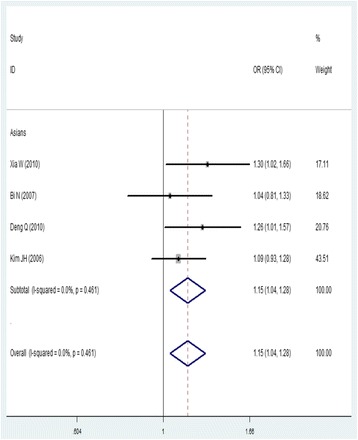
Forest plot of the association between rs664143 and lung cancer risk (A vs. G). OR: odds ratio, CI: confidence interval

For *ATM* rs609429 (98,158 G > C), we identified 1490 cases and 1501 controls in three studies. We failed to find any significant associations (Fig. 4; Table 3).

**Fig. 5:**
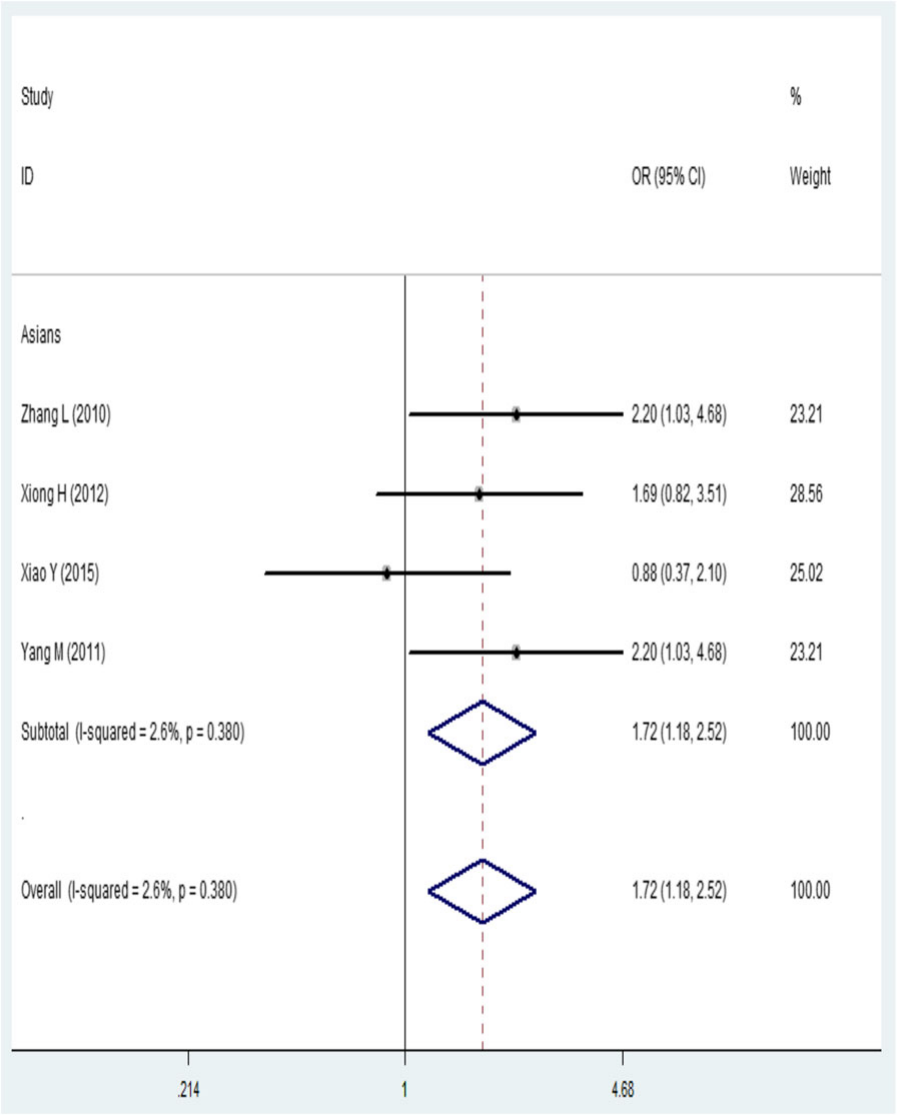
Forest plot of the association between rs189037 and RP risk (AA + AG vs. GG). OR: odds ratio, CI: confidence interval

With regard for the association between the risk of RP and the *ATM* rs189037 polymorphism, we identified four studies that contained 192 cases and 772 controls. The results showed that there was a significant association between this SNP and the risk of RP (AA/AG versus GG, OR = 1.72, 95% CI = 1.18–2.52; AA versus GG, OR = 1.74, 95% CI = 1.02–2.97) (Fig. 5; Table 3). No publication bias was detected in a funnel plot or Egger’s test (*P =* 0.303).

## Discussion

In the present meta-analysis, we found that there were significant associations between lung cancer risk and rs189037, rs664677 and rs664143. Additionally, we found that the rs189037 A allele was significantly associated with the risk of RP. Significant heterogeneity was found in two of the genetic models used to evaluate the risk of RP. This may be because different diagnostic standards were used in different studies. Fortunately, the genetic models identified in our study that did produce significant associations results showed no significant heterogeneity. No significant publication bias was found in polymorphisms that were covered by at least five studies.

*ATM* rs189037 is located in the 5’UTR of the promoter region of the ATM gene [36]. This SNP can bind to the activator protein 2α (AP-2α), which represses *ATM* transcription, and different genotypes have different affinities for this transcription factor [37]. As some studies have suggested, the AA genotype of *ATM* rs189037 increases the risk of oral [38] and breast cancer [39]. Furthermore, this mutation may be associated with the risk of thyroid carcinoma [40]. *ATM* rs664143 is located in the protein binding motifs that serve as binding sites for intronic splicing enhancers or repressors, suggesting the possibility that this site may be involved in the exon 61 splicing process and that it may cause inaccurate splicing [24]. The precise roles of rs664677 and rs609429 remain unclear [41]. The former loci may participate in regulating RNA splicing and maintaining RNA stability [41]. A study performed in Korea showed that the rs664677 CC genotype might be associated with a higher risk of breast cancer [42]. However, rs664677 was not associated with either papillary thyroid carcinoma [43, 44] or pancreatic cancer [45]. Other studies failed to find a significant association between this loci and the risk of cancers, such as breast cancer [46, 47]. DNA repair pathways are activated after DNA damage, especially DNA DSBs [20]. Individuals with impaired DNA repair pathways have high sensitivity to radiation exposure and therefore a higher risk of lung cancer and RP [8, 20]. Previous studies have suggested that the *ATM* gene plays a critical role in DNA damage repair and thereby affects the risk of lung cancer and RP. We performed this meta-analysis because the results of previous studies were inconclusive.

Several limitations of the current analysis should be mentioned. First, we failed to conduct a subgroup analysis to evaluate the effects of other factors, such as gender, smoking status or histological subtypes, because insufficient data was available. Second, we included only articles published in either English or Chinese, resulting in potential publication bias, despite the fact that the Egger’s test and funnel plots showed that there was no publication bias. Despite these limitations, the likelihood of bias was minimized throughout the process because we used a detailed protocol that included study identification, data selection and the statistical analysis, and we controlled publication bias. Therefore, we believe that our results are reliable. Additionally, most polymorphisms and studies of the *ATM* gene are included in this study. It is also the first meta-analysis to evaluate the association between *ATM* gene polymorphisms and the risk of RP.

## Conclusions

In conclusion, in this meta-analysis, we showed that the *ATM* rs189037 polymorphism is significantly associated with lung cancer and the risk of RP after radiotherapy. The *ATM* rs664677 and *ATM* rs664143 polymorphisms were significantly associated with lung cancer susceptibility, while rs609429 was not. More well-designed studies that include larger sample sizes should be performed in the future to further evaluate the association between the *ATM* gene and cancers.

## Abbreviations

ATM: Ataxia telangiectasia mutated
BRCA1: Breast cancer protein 1
CHK2: Cycle checkpoint kinase 2
CI: Confidence intervals
CNKI: China National Knowledge Infrastructure
DNA: Deoxyribonucleic acid
DSB: Double-strand breaks
MDM2: Murine double minute 2
NBS1: Nijmegen breakage syndrome
OR: Odds ratios
RNA: Ribonucleic acid
RP: Radiation-induced pneumonitis
SNP: Single nucleotide polymorphism
